# High-Performance Aramids with Intrinsic Bactericide Activity

**DOI:** 10.1021/acsami.3c17919

**Published:** 2024-02-07

**Authors:** Sandra de la Parra, Álvaro Miguel, Natalia Fernández-Pampín, Carlos Rumbo, José M. García, Ana Arnaiz, Miriam Trigo-López

**Affiliations:** † Departamento de Química, Facultad de Ciencias, Universidad de Burgos, Plaza de Misael Bañuelos s/n, 09001 Burgos, Spain; ‡ International Research Center in Critical Raw Materials for Advanced Industrial Technologies (ICCRAM), R&D Center, Universidad de Burgos, Plaza de Misael Bañuelos s/n, 09001 Burgos, Spain; § Universidad Politécnica de Madrid, Calle Ramiro de Maeztu, 7, 28040 Madrid, Spain; ∥ Facultad de Ciencias, Universidad Autónoma de Madrid, Calle Francisco Tomás y Valiente 7, Fuencarral-El Pardo, 28049 Madrid, Spain

**Keywords:** aramids, high-performance polymers, advanced functionalities, bactericide, textile

## Abstract

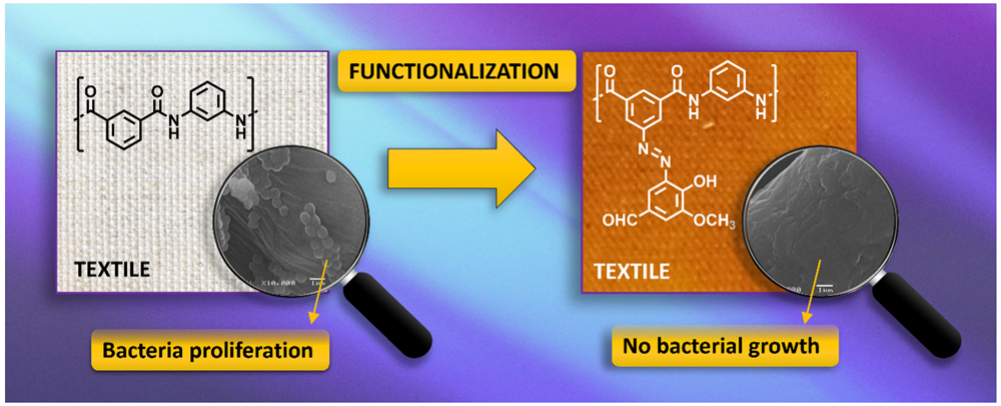

Aramids, renowned for their high-performance attributes, find applications in critical fields such as protective equipment, aerospace components, and industrial filters. However, challenges arise in scenarios in which frequent washing is impractical, leading to bacterial proliferation, especially in textiles. This study outlines a straightforward and scalable method for preparing aramid-coated textiles and films endowed with inherent bactericidal activity, achieved by reacting parent aramids with vanillin. The functionalization of the aramids with bactericide moieties not only preserved the high-performance characteristics of commercial aramids but also improved their crucial mechanical properties. Tensile tests revealed an increase in Young’s modulus, up to 50% compared to commercial *m*-aramid, accompanied by thermal performance comparable to commercial *m*-aramids. The evaluation of these coated textiles as bactericidal materials demonstrated robust effectiveness with *A* parameters (antibacterial activity) of 4.31 for *S. aureus* and 3.44 for *K. pneumoniae*. Reusability tests (washing the textiles in harsh conditions) underscored that the bactericide-coated textiles maintain their performance over at least 5 cycles. Regarding practical applications, tests performed with reconstructed human epidermis affirmed the nonirritating nature of these materials to the skin. The distinctive qualities of these metal-free intrinsic bactericidal aramids position them as ideal candidates for scenarios demanding a synergy of high performance and bactericidal properties. Applications such as first responders’ textiles or filters stand to benefit significantly from these advanced materials.

## 1 Introduction

Aromatic polyamides, also known as aramids, are a class of high-performance polymers that are renowned for their remarkable thermal and mechanical properties and their chemical resistance. The commercial and most well-known aramids are poly(*p*-phenylene teterphthalamide) (PPTA) and poly(*m*-phenylene isophthalamide) (MPIA). These polymers possess the advantage of being lightweight while exhibiting exceptional strength, allowing them to be utilized in various forms, such as dense or porous membranes, coatings, and textiles.^1^ Aramids find applications in aerospace, automotive, and sports industries, protective equipment and garments, and filtration applications. Their unique properties of strength, heat resistance, chemical resistance, and durability make them sought-after materials in industries where these factors are crucial.^2^ As textiles, they are highly versatile and therefore used for various industries. They are employed in protective clothing for the military, law enforcement, and firefighters as well as for ballistic protection (bulletproof vests) and industrial safety gear, including gloves, sleeves, aprons, or footwear.

Due to their extensive surface area and moisture retention capacity, textiles can become breeding grounds for microorganisms. Pathogenic microorganisms pose a risk to human health, significantly impacting health facilities and food safety. Regular laundering is the most viable and effective method to eliminate or minimize microbial presence on textiles.^3^ However, some fabrics cannot be washed frequently, are rarely exposed to light, and are sometimes stored for extended periods and at high moisture levels, creating an ideal environment for microbial proliferation. Nevertheless, an alternative approach to mitigating microbial infection risks is the development of antimicrobial or biocide fibers, which is gaining attention, especially after the SARS-CoV-2 pandemic. Ideally, these antimicrobial materials should effectively destroy, deter, render harmless, or exert control over a wide range of pathogenic microorganisms without exhibiting any harmful effects on the skin.^4^ Moreover, striking a balance between the antimicrobial efficacy and skin safety is essential for the optimal design of such fibers or textiles.

Aramid textiles, especially those used for military and protective clothes, lack antimicrobial properties and cannot be frequently washed. Moreover, emergency responders have an increased risk of exposure to biological pathogens^5^ since they provide additional emergency medical service responses without adequate protection against biological agents.^6^ Traditionally, the use of antimicrobial nanoparticles, particularly silver nanoparticles, has been demonstrated to be a promising strategy against microorganism growth in textiles, and some examples of their use in aramids can be found in the literature.^7−10^ However, they tend to agglomerate, impairing a good dispersion in the textile, and some studies have indicated that silver nanoparticles can be released from textiles during washing, leading to environmental bioaccumulation, increased human exposure to silver, and losing antimicrobial activity in the textiles.^11,12^

Another simple and effective way to provide *m*-aramids with antimicrobial properties is to prepare *N*-halamines by chlorination of the nitrogen in the amide group. Lee and co-workers performed this strategy for the preparation of filtration membranes made of aramids,^13^ or aramids blends with other polymers like cellulose, polyethylene terephthalate (PET), polyacrylonitrile (PAN), or poly(vinyl alcohol) (PVA).^14−18^ The biocidal mechanism of *N*-halamine involves its direct contact with the microbial cell, followed by the transfer of the oxidative halogen into the cell. Since the oxidative halogen is lost, the material must be recharged (chlorinated again) to restore its antimicrobial effectiveness, which can be a drawback, particularly in the mentioned textiles.^19^ Furthermore, the high crystallinity of aramid fabrics in textiles poses challenges to the efficiency of the chlorination process, while the interchain interactions through hydrogen bonding of the amide link, and therefore their high-performance properties, are lost when clorinated.^6^

An alternative strategy to provide aramids with antimicrobial properties is the functionalization with specific groups. This approach has also been carried out with aramids, by preparing aramid dendrimers with amine-end groups,^20^ cysteine residues,^21^ quaternary ammonium cation and salicylaldehyde,^22^ or sulfonamidopyrimidines pendant structures groups.^23^

Recently, vanillin (4-hydroxy-3-methoxybenzaldehyde), which can be extracted from vanilla pod, has been used to prepare ecofriendly or biobased polymers with antimicrobial purposes. However, it possesses a small chemical structure and leaches to the media if it is not chemically anchored to the polymer or textile.^24−26^ Vanillin demonstrates antimicrobial activity against a number of yeasts, molds, and bacteria by affecting the integrity of the cytoplasmic membrane and inhibiting respiratory activity.^27^

**Figure 1:**
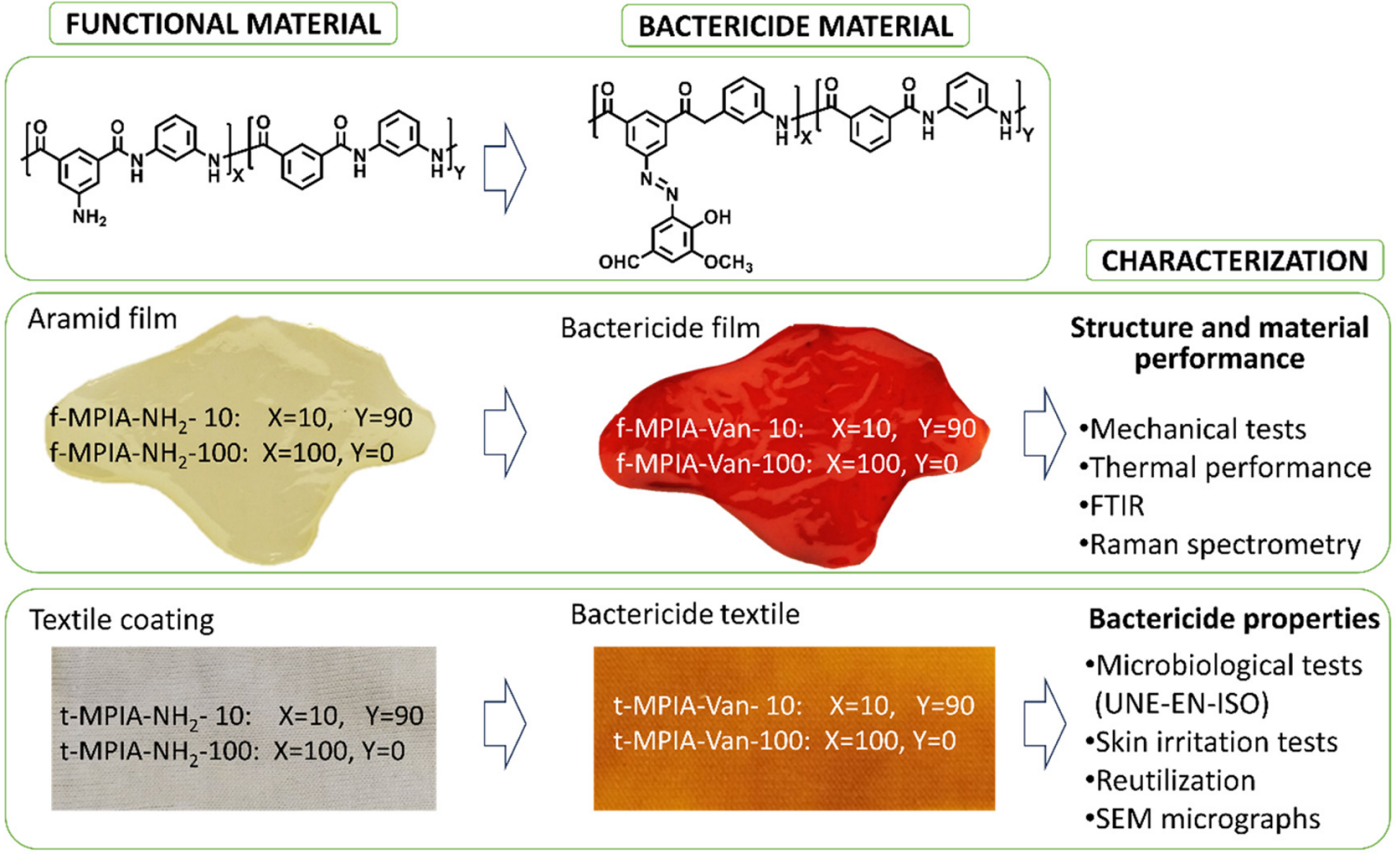
Methodology for the preparation and characterization of the bactericide materials.

This work aims to prepare aramids with antimicrobial properties to be used in textiles using vanillin as a natural product. We used our previously designed and prepared aramids containing reactive amino groups.^28,29^ These free amino groups can be easily modified through solid-phase azo-coupling reactions in water to anchor the vanillin to the aramid (Figure 1). The procedure could be easily scaled up to prepare aramid fibers containing vanillin through a post-treatment on aramid fibers containing amino groups. To simulate this, we prepared amino-functionalized aramid coatings on cotton textiles and performed azo-coupling on them. At the same time, we prepared aramid films containing free amino groups and reacted them with vanillin to characterize the new materials.

The uniqueness of our antimicrobial material lies in its ability to produce textiles that possess a range of specific properties and characteristics. First, the textile is free from any metal content. Second, the antimicrobial agent is chemically bonded to the polymer, ensuring that the material retains its effectiveness even after repeated use or washing. Third, the preparation procedure is scalable, allowing for large-scale production. Fourthly, they are nonirritant to the skin, which makes them adequate skin contact textiles. Lastly, the combination of exceptional tensile strength, heat resistance, and lightweight nature makes them efficient for filtration applications and reliable for tasks such as projectile containment, impact absorption, and thermal insulation in high-temperature environments, protective equipment, and clothing.

## 2 Materials and Methods

### 2.1 Materials

All materials and solvents used in this work are commercially available and were used as received unless otherwise specified: 5-aminoisophthalic acid (Sigma-Aldrich, 94%), *N*,*N*-dimethylformamide (DMF, Merck, >99%), sodium nitrite (NaNO_2_, Panreac, >98%), sodium azide (NaN_3_, Alfa Aesar, 99%), thionyl chloride (SOCl_2_, Thermo Scientific, 99,7%), vanillin (Thermo Scientific, 99%), sodium hydroxide (NaOH, VWR Chemicals, 99%), hydrochloric acid (HCl, VWR Chemicals, 37%), sodium borohydride (NaBH_4_, Alfa Aesar, 98%). *N*,*N*-Dimethylacetamide (DMA, VWR Chemicals, >99%) was vacuum-distilled twice over phosphorus pentoxide (P_2_O_5_, Alfa Aesar, 98%) and then stored in the presence of 4 Å molecular sieves. *m*-Phenylenediamine (MPD, Sigma-Aldrich, 99%) was purified by double-vacuum sublimation and stored in a nitrogen atmosphere. Lecithin from egg (TCI), Bacto tryptone pancreatic digest of casein (Gibco), NaCl (Labkem), soya peptone (enzymatic digest of soybean meal) (VWR), polysorbate 80 (VWR), l-(+)-histidine monohydrochloride monohydrate (VWR, 98%), d-glucose anhydrous (Fisher Chemical, 99%), Difco agar (BD), potassium phosphate monobasic (Sigma, 99%), disodium hydrogen phosphate anhydrous (Panreac, 99%), dipotassium hydrogen phosphate (Supelco, ≥99%).

The *in vitro* EpiDerm skin irritation test (EPI-200-SIT) utilized tissues and reagents supplied by MatTek In Vitro Life Science Laboratories. These included EpiDerm tissues (EPI-200-SIT), DMEM medium (EPI-100), a 5% solution of sodium dodecyl sulfate (SDS) (TC-SDS-5%), and the MTT-100 assay kit (MTT-100).

The cotton textile (t-Control) was obtained from a 100% cotton t-shirt purchased from Decathlon.

### 2.2 Methods

An FT/IR-4200 Jasco spectrometer with an ATR-PRO410-S single reflection accessory was used to record the polymers’ infrared spectra (FT-IR).

Raman spectra were acquired through a confocal AFM-Raman system, namely, the Alpha300R–Alpha300A AFM (WITec). The experiments utilized laser radiation at 785 nm with a power output of 2 mW, employing a magnification of 100×. The thermal behavior of the aramid films was evaluated by thermogravimetric analysis (TGA) using about 5 mg of samples in a TGA Q50 TA Instruments thermobalance. The measurements were performed under both nitrogen and synthetic air at a heating rate of 10 °C/min. The char yield (CR) value at 800 °C under a nitrogen atmosphere was used to calculate the limiting oxygen index (LOI) using LOI = 17.5 + 0.4 CR.^30^

Differential scanning calorimetry (DSC) experiments were conducted by employing a Q200 TA DSC apparatus from TA Instruments, utilizing samples of about 10 mg. Initially, the sample underwent a heating process at a rate of 15 °C/min, starting from room temperature and reaching 350 °C. Subsequently, it was maintained at this temperature for 5 min to erase the thermal history of the polymer. Following this, the sample was cooled to −80 °C at a rate of 15 °C/min, heated again to 350 °C at a rate of 10 °C/min to investigate the thermal transitions, and finally cooled to −80 °C at a rate of 10 °C/min.

Water uptake experiments were performed gravimetrically using a TGA Q50 TA Instruments thermobalance. The film samples were vacuum-died overnight at 80 °C (20–25 mg samples) and then kept in a 65% relative humidity environment for 1 week. The 65% relative humidity environment is achieved by placing the samples in a closed box at 20 °C containing a concentrated solution of NaN0_2_. After the week, the samples were placed in the TGA and the weight loss was recorded while heated to 100 °C at 10 °C/min and maintained at that temperature for 15 min.

The mechanical performance of the aramid films was tested using a Shimazdu EZ Test Compact Table-Top Universal tester, 5 mm × 30 mm cut strips, and subjected to an extension rate of 5 mm/min with a 9.44 mm gauge length. At least five tests are performed for each sample; the highest and lowest values are eliminated, and the rest are then averaged.

Scanning electron microscopy (SEM) images were obtained with a JEOL JSM-6460LV scanning electronic microscope. The textile samples were coated with gold after achieving the standard UNE-EN-ISO 20743:2022 in t-Control (cotton textile) and t-MPIA-Van-100 textiles. In addition, t-Control and t-MPIA-Van-100 samples without treatment were included.

### 2.3 Preparation of Aramid Films and Coatings on Textiles

Two aramids containing 10% and 100% of free amino groups (functional polymers, MPIA-NH_2_-10 and MPIA-NH_2_-100, respectively) were prepared following the previously described procedure.^29^

Functional aramid films (f-MPIA-NH_2_-10 and f-MPIA-NH_2_-100) were prepared following the common solution-evaporation (casting) procedure: 0.35 mg of the amino-containing aramid is dissolved in 5 mL of DMA. The solution is filtered off and poured on a glass plate inside an air-circulating oven. The temperature is kept at 80 °C for 24 h to remove the solvent. The obtained films are then washed carefully with water to remove any solvent traces before reacting with vanillin.

Functional aramid coatings on cotton textiles (t-MPIA-NH_2_-10 and t-MPIA-NH_2_-100) were prepared following the drop-coating procedure: 0.203 mg of the polymer is dissolved in 5.7 mL of DMA. The solution was poured drop by drop on an 80 mm × 55 mm washed 100% cotton textile (cut from a t-shirt), while the solvent was evaporated inside an air-circulating oven at 80 °C for 24 h. The coated textiles were washed with water before reacting with vanillin. Additionally, a MPIA coated textile (t-MPIA) was prepared in the same fashion.

### 2.4 Preparation of the Antimicrobial Films and Coatings on Textiles

The functional aramid coated textiles (t-MPIA-NH_2_-10 and t-MPIA-NH_2_-100) were immersed in 50 mL of an aqueous solution containing 5 mL of HCl and 200 mg of NaNO_2_ overnight. Then, the textiles were washed with water and immersed overnight in an aqueous solution containing 24 mL of 1 M NaOH, 16 mL of methanol, and 300 mg of vanillin. The antibacterial-coated textiles (t-MPIA-Van-10 and t-MPIA-Van-100) are washed with hot water and ethanol and air-dried. The antimicrobial films (f-MPIA-Van-10 and f-MPIA-Van-100) are prepared in the same fashion using the functional aramid films (f-MPIA-NH_2_-10 and f-MPIA-NH_2_-100) and twice the amount of the mentioned solutions.

### 2.5 Bacterial Strains and Inoculum Preparation

In this research, *Staphylococcus aureus* WDCM 00193 (ATCC 6538, CECT 239) and *Klebsiella pneumoniae* WDCM 00192 (ATCC 4352, CECT 8453) were used as indicated by UNE-EN-ISO 20743:2022. Both bacteria were maintained in tryptocasein soy agar (TSA). One colony of each bacteria was inoculated into tryptone soy broth (TSB) and growth at 180 rpm at 37 °C overnight to attain a viable cell concentration ranging from 1 × 10^8^ to 3 × 10^8^ CFU/mL. Subsequently, 0.4 mL of initial inoculum was diluted in 20 mL of TSB medium and incubated for approximately 3 h under optimal growth conditions to obtain a bacterial concentration of 10^7^ CFU/mL.

### 2.6 Antimicrobial Tests

The objective of the assay was to assess the antibacterial capacity of the textile fabrics described above against *S. aureus* and *K. pneumoniae* bacteria following the absorption method described by UNE-EN ISO 20743:2022, with minor modifications.

The textiles tested were cut into rectangular shapes (2 cm × 1 cm). After autoclave sterilization (121 °C, 20 min, 1 atm), 200 μL of the previously prepared bacterial culture was homogeneously distributed on each textile. Then, the textiles with an exposure time of 0 h were extracted with 3.5 mL of neutralizer solution (as indicated by the standard with 50 g/L of polysorbate 80) using vortex for 1 min and sonicated in an ultrasonic bath for 20 min in order to improve bacterial extraction. After this step, suspensions were serially diluted and plated on a TSA medium to determine the number of viable cells, and they were incubated for 24 h at 37 °C. In addition, the textiles exposed for 24 h were incubated at 37 °C immediately after inoculation. At the end of this time, the textiles were transferred to new test vials, where extraction and seeding were performed by following the method described above. Each assay included three independent replicates.

The antibacterial activity of textile products was assessed by comparing the number of viable bacteria following incubation with the treated and control textile products. The inhibition percentage and *A* parameter were determined using the following eqs 1 and 2:
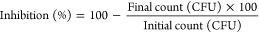
1
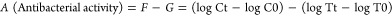
2where *F* = bacteria growth value obtained on the control textile sample; log Ct = decimal logarithm of the arithmetic mean of the bacterial count obtained from three control samples after a 20 h incubation; log C0 = decimal logarithm of the arithmetic mean of the bacterial count obtained from three control samples immediately after bacterial inoculation; *G* = bacteria growth value obtained on the textile sample with antibacterial treatment; log Tt = decimal logarithm of the arithmetic mean of the bacterial count obtained from three textile samples with antibacterial treatment after a 20 h incubation; log T0 = decimal logarithm of the arithmetic mean of the bacterial count obtained from three textile samples with antibacterial treatment immediately after bacterial inoculation.

### 2.7 Washing Operation

After carrying out the antimicrobial tests, the samples were subjected to a washing protocol. First, the samples were washed three times with 70% ethanol for 16 h, applying vortex agitation after each wash. Subsequently, a wash was applied for 16 h with a 2% Tween-20 solution to eliminate possible residues adhered to the fabric. They were then washed twice with water and finally washed with acetone. The samples were allowed to dry and were autoclaved before being used again. This protocol was carried out between each antimicrobial test to evaluate the reusability of the material at least five times.

### 2.8 *In Vitro* Skin Irritation Test

The skin irritation potential of t-MPIA-Van-100 and a cotton textile (t-Control, as control material) was evaluated by an *in vitro* EpiDerm skin irritation test (EPI-200-SIT, MatTek In Vitro Life Science Laboratories, 2020).

Upon receipt, the tissues were examined for any damage, following the manufacturer’s guidelines. Subsequently, to mitigate the stress related to the transport, they were placed in 6-well plates containing 0.9 mL of assay medium (EPI-100-NMM) and incubated under optimal conditions (37 ± 1 °C, 5% ± 1% CO_2_, 90% ± 10% RH) for 1 h. Then, they were transferred to a freshly prepared medium and left to incubate overnight (18 ± 3 h) under optimal conditions. Following this, the tissues underwent exposure to the textiles t-Control and t-MPIA-Van-100 for 1 h. Tissues treated with DPBS (25 μL) were used as negative control, while tissues exposed to 5% SDS (25 μL) were considered as positive control. Each test material and control were tested on three separate tissue samples.

After exposure, the tissues were rinsed 15 times with DPBS, following the manufacturer’s guidelines, and they were transferred to a 6-well plate with 0.9 mL of culture medium. Then, the samples were incubated under optimal conditions for 24 ± 2 h. After this period of time, the culture medium was replaced with fresh medium and the tissues were incubated again for 18 ± 2 h under optimal conditions.

Following the procedures outlined in OECD Guideline Test No. 439, the effects of the exposure to the textiles on the tissue viability was evaluated by MTT assay. After the 18 ± 2 h of incubation, the tissues were placed in a 24-well plate containing 0.3 mL of an MTT solution at 1 mg/mL and incubated for 3 h under optimal conditions. Subsequently, the tissues were washed twice with DPBS, and the formazan crystals were solubilized by adding 2 mL of isopropyl alcohol (MTT-100-EXT) and agitating for 2 h at room temperature. After the extraction time, the tissues were pierced with an injection needle, allowing the extract to flow into the well from which the inset was taken, and then, they were discarded, and the extraction solutions were mixed and transferred to a 96-well plate.

Tissue viability was determined by measuring the optical density (OD) at 570 nm of each sample extract in duplicate by using a plate reader (BioTek Synergy HT). Isopropanol was used as a blank. The tissue viability percentage was calculated relative to the negative control using the following eq 3:
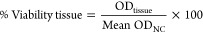
3

### 2.9 Statistical Analyses

Statistical assessments were conducted using GraphPad Prism v8. Initially, an examination was carried out to assess data normality and homoscedasticity. Upon confirming the fulfilment of both assumptions, a one-way ANOVA was executed, followed by Tukey’s multiple comparisons tests (*p* ≤ 0.05).

## 3 Results and Discussion

### 3.1 Preparation of Antibacterial Model Aramid, Films, and Coatings

The preparation of functional aramids containing amino groups as parent functional materials was described in previous work.^29^ In that work, we described the synthesis of those materials containing amino groups for the preparation of many others in an easy manner. So, this time, we prepared antibacterial materials from them. Since the functionalization of these parent functional materials with antibacterial moieties can only be performed in an aqueous solution, we prepared films and coated textiles with functional polymers (MPIA-NH_2_-10 and MPIA-NH_2_-100), as described in section 2.3, to perform the reactions in the solid state.

**Figure 2:**
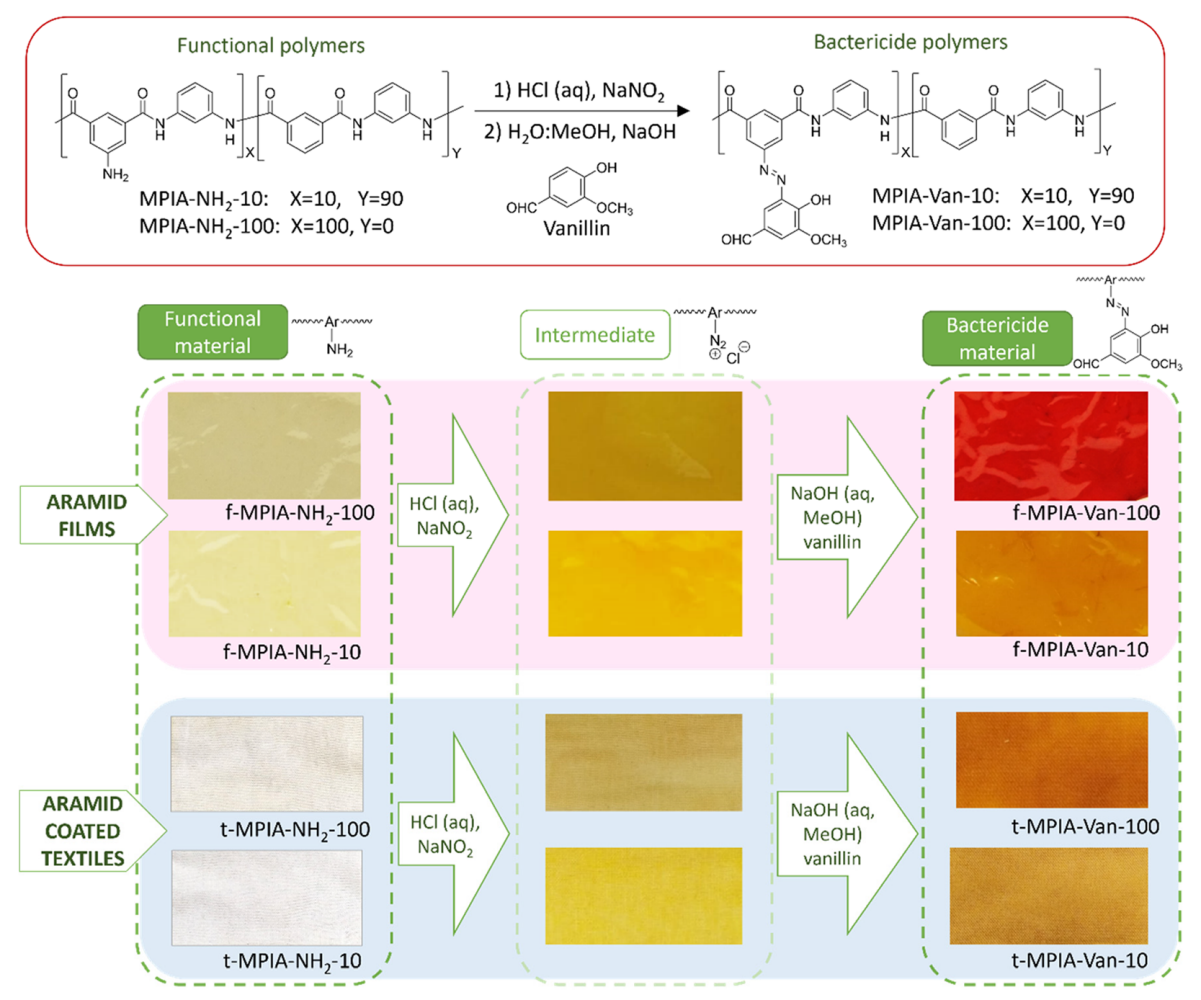
Functionalization reaction of the functional polymers with vanillin moieties to obtain bactericide polymers, with color evolution during the reaction steps in the films and coated textiles.

The functionalization with vanillin (Figure 2) is performed through an azo-coupling reaction. First, the diazonium salt is formed in the films and coated textiles by immersing them in an acidic solution of NaNO_2_ when the materials turn yellow. Then, the azo coupling is formed with the activated aromatic ring of vanillin, which can be observed as the material’s color turns red due to the formation of the azo (−N=N−) group (Figure 2). The new antibacterial polymers are insoluble even in polar aprotic solvents (Table 1), probably due to cross-linking, impairing their characterization using solution nuclear magnetic resonance (NMR). The reaction can be followed visually by the color transformation both in the films and in the coated textiles. However, FTIR spectroscopy is a key technique for monitoring these reactions since the formation of the diazonium salt (−N_2_^+^Cl^–^) is evidenced by the emergence of a tension band at 2278 cm^–1^, which later disappears when the azo group is formed.^26^Figure S1 in the Supporting Information, section S1, illustrates the FTIR spectra of the functional film (f-MPIA-NH_2_-100), the formation of the diazonium salt in the film (f-MPIA-N_2_^+^Cl^–^), and the bactericide film (f-MPIA-Van-100). The reaction was also followed by Raman spectroscopy on the surface of the films. The modification of the chemical structure of the functional materials is verified by the formation of azo group (stretching band around 1400 cm^–1^) and the appearance of the −OCH_3_ stretching band (around 1300 cm^–1^) (Supporting Information, section S1, Figure S2).^31^ This way, we prepared two antibacterial textiles (t-MPIA-Van-10 and t-MPIA-Van-100) and two antibacterial films (f-MPIA-Van-10 and f-MPIA-Van-100). The antibacterial films were used to characterize the new materials as polymers, while textiles were used to characterize their antibacterial performance.

**Table 1:** Water Uptake and Solubility of the Polymer Films and Vanillin

	water uptake^a^	solubility^b^				
polymer	mass (%)	DMA	DMF	DMSO	NMP	EtOH, THF, acetone, CH_2_Cl_2_, CHCl_3_
Vanillin	na	++	++	++	++	++
f-MPIA	6.7	++	++	++	++	–
f-MPIA-NH_2_-10	7.8	++	++	++	++	–
f-MPIA-NH_2_-100	9.3	++	++	++	++	–
f-MPIA-Van-10	8.5	–	–	–	–	–
f-MPIA-Van-100	10.5	–	–	–	–	–

a RH = 65%, *T* = 20 °C.

b 10 mg of polymer/1 mL of solvent; ++ = soluble at room temperature; + = soluble on heating; +– = partially soluble; – = insoluble.

The films possess a width of approximately 30 μm. Given that the reaction with vanillin is conducted in a water-based environment, it is likely that only the surface-exposed functional groups have undergone a reaction with vanillin within the films. In contrast, the coated textiles offer a significantly larger surface contact area. Consequently, it is plausible that a substantial portion of the amine-containing aramid within textiles has been functionalized with vanillin.

### 3.2 Characterization of the Antibacterial Polymers

The functional and bactericide films were characterized in terms of solubility, water uptake, and thermal and mechanical performance and compared to the MPIA film synthesized under the same conditions.

Both MPIA and the functional films containing amino groups (f-MPIA-NH_2_-10 and f-MPIA-NH_2_-100) are soluble only in polar aprotic solvents (Table 1), as expected for aramids, while the new bactericide polymers f-MPIA-Van-10 and f-MPIA-Van-100 are insoluble in any solvents, demonstrating the cross-linking of the materials when vanillin is introduced in the structure.

Water sorption can enhance some properties of aramids and at the same time prejudice others. It can worsen their mechanical and thermal performances, while it is beneficial for applications related to their use as filtration membranes. Water uptake in the films is associated with the presence of polar groups in the material’s structure. In this way, aramids with -NH_2_ groups absorb a larger amount of humidity than the commercial aramid, and therefore, aramids functionalized with vanillin absorb even more.

The thermal performance of aramid functional and bactericide polymer films was characterized both in nitrogen atmosphere and synthetic air using thermogravimetric analysis in terms of T_5_, T_10_, char yield, and limiting oxygen index (LOI) and compared to commercial MPIA. Also, the thermal transitions of the materials were evaluated using DSC (Table 2 and Supporting Information, section S2, Figure S3 and Figure S4). The *T*_g_ of the functional materials with 10% amino groups is very similar to that of MPIA. This *T*_g_ value is increased to 301 °C with 100% amino groups present in the aramid due to increased interactions between polymer chains. The *T*_g_ of the material containing vanillin is increased compared to the commercial MPIA, caused by the partial cross-linking of the material and the fact that the larger volume of vanillin lateral moiety impairs chain mobility. However, no *T*_g_ was observed for f-MPIA-Van-100, demonstrating the larger cross-linking of the material containing 100% vanillin groups.

**Table 2:** *T*_g_ and Thermal TGA Data of the Films under a Nitrogen Atmosphere and Synthetic Air

		nitrogen atmosphere			synthetic air atmosphere			
polymer film	*T*_g_ (°C)	*T*_5_^a^ (°C)	*T*_10_^b^ (°C)	char yield (%)	*T*_5_^a^ (°C)	T_10_^b^ (°C)	char yield (%)	LOI^c^
MPIA film^d^	273	448	461	51.0	447	464	1.1	38
f-MPIA-NH_2_-10	279	421	439	50.9	415	445	0.8	38
f-MPIA-NH_2_-100	301	413	436	55.1	434	477	0.9	40
f-MPIA-Van-10	283	417	441	61.2	409	446	0.9	42
f-MPIA-Van-100		364	445	61.3	334	449	2.7	42

a 5% weight loss temperature (*T*_5_), 10% weight loss temperature (*T*_10_).

b At 800 **°**C.

c Limiting oxygen index, calculated from the TGA data^30^ (LOI = 17.5 + 0.4 CR, where CR is the char yield in % weight at 800 °C).

d MPIA film prepared following the same procedure.

Regarding the thermogravimetric analysis, a slight decrease in the *T*_5_ and *T*_10_ compared to MPIA film is observed in the materials both in synthetic air and under a nitrogen atmosphere. This fact is correlated to the breakdown of the non-cross-linked lateral groups in the aramid structure (−NH_2_ or vanillin) as temperature increases, which is more evident in f-MPIA-Van-100. However, the char yield (%) under nitrogen atmosphere increases for vanillin-containing materials compared to MPIA, probably due to cross-linking, resulting in an increase in the LOI of the material up to 42.

The mechanical performance of the films is summarized in Table 3. Both the functional and bactericide films showed an increase in Young’s modulus compared to commercial MPIA films. Functional films possess free amino groups in the main chain, enabling interactions with other chains through hydrogen bonding, thus increasing the films’ rigidity, which is especially evident in the film containing more amino groups (f-MPIA-NH_2_-100). On the other hand, and as mentioned before, the films cross-link with the reaction with vanillin, giving rise to films with improved Young’s modulus. As a result of this increase in the rigidity of the films, the elongation at the break diminishes, as expected. The tensile strength of the films is well compared to that of MPIA, showing no significant variation related to the presence of amino groups or vanillin.

**Table 3:** Mechanical Performance of the Prepared Films

polymer film	Young’s modulus (MPa)	tensile strength (MPa)	elongation at break (%)
MPIA film^a^	1271 ± 56	72 ± 9	32 ± 11
f-MPIA-NH_2_-10	1462 ± 205	72 ± 6	33 ± 8
f-MPIA-NH_2_-100	2060 ± 76	64 ± 6	8 ± 2
f-MPIA-Van-10	1709 ± 174	81 ± 3	15 ± 1
f-MPIA-Van-100	1896 ± 150	71 ± 5	5 ± 1

a MPIA film prepared following the same procedure.

### 3.3 Antibacterial Activity of the Textiles

The antibacterial tests were performed using *S. aureus* and *K. pneumoniae*, as indicated by the standard. Both bacterial strains belong to the ESKAPE group of microorganisms due to the ease with which they develop antibiotic resistance, mainly in hospital infections.^32^Table 4 shows the antibacterial efficiency of the tested materials expressed as *A*, which should be greater than or equal to 2 to consider that the material has significant antibacterial properties, as expressed in the standard. The tests were performed with textiles coated with the parent polymers since some works also reported amino groups (−NH_2_) to show some bactericide effect.^33,34^ The results were also compared to commercial MPIA coated textile (t-MPIA). As a result of the experiments carried out, all the experimental data obtained for each tissue are detailed in Supporting Information, section S3 and Tables S1–S5. Consequently, only t-MPIA-Van-100 can be considered an antibacterial material since its *A* value is greater than 2, presenting a strong antibacterial effect according to the standard (greater than or equal to 3) for both bacterial strains, *S. aureus* and *K. pneumoniae*. However, t-MPIA-Van-10 also presents a statistically significant effect with both bacterial strains, but the effectiveness of the antibacterial properties is considered low according to the standard. These results demonstrate the positive effect of more vanillin groups anchored to the polymer on the obtention of bactericide materials. A higher water uptake in materials could be considered a drawback, particularly considering that wet environments often encourage bacterial proliferation. Nevertheless, f-MPIA-Van-100 exhibits the greatest water uptake among all of the tested materials but also emerges as the most potent antibacterial material, thereby emphasizing the notable impact of vanillin groups.

**Table 4:** Analyses of Textiles’ Antibacterial Capacity and Efficacy (*A* Parameter) (UNE-EN ISO 20743:2022)^a^

	*S. aureus* WDCM 00193	*K. pneumoniae* WDCM 00192
t-MPIA	–0.04 ± 0.07 c	0.23 ± 0.02 c
t-MPIA-NH_2_-10	–0.04 ± 0.02 c	0.19 ± 0.05 c
t-MPIA-NH_2_-100	0.15 ± 0.01 c	0.35 ± 0.10 c
t-MPIA-Van-10	1.30 ± 0.18 b	0.87 ± 0.07 b
t-MPIA-Van-100	4.31 ± 0.20 a	3.42 ± 0.19 a

a Data are mean values ± SE of three independent experiments. Different letters denote significant differences within each bacterial species. One-way ANOVA, *p* ≤ 0.05, followed by Tukey’s multiple comparisons test.

### 3.4 Reuse of Antibacterial Textile Material

The antibacterial textile, t-MPIA-Van-100, underwent up to five cycles of cleaning and reusing. The assessment of its antibacterial efficacy against *S. aureus* and *K. pneumoniae* microorganisms, was performed as delineated in section 2.7. The laundering and sterilization procedures were executed per the guidelines provided in section 2.8, aimed at eradicating any residual bacterial contaminants. Upon these reuses, a slight decrease in parameter A was observed in both assays; however, this reduction did not attain statistical significance. Remarkably, the material maintained significant antibacterial activity and effectiveness, conforming to established standards (Table 5). Furthermore, the growth inhibition percentages were 99.43 ± 0.27 and 99.26 ± 0.64 for *S. aureus* and *K. pneumoniae*, respectively, following the fifth cycle of use, indicating that textile t-MPIA-Van-100 preserved high and robust antibacterial activity against both microorganisms even when subjected to drastic cleaning conditions.

**Table 5:** Antibacterial Capacity and Efficacy (UNE-EN ISO 20743:2022) of t-MPIA-Van-100 Textile after 5 Cycles of Cleaning and Reusing^a^

	*S. aureus* WDCM 00193		*K. pneumoniae* WDCM 00192	
cycle	antibacterial activity (*A*)	% growth inhibition (CFU)	antibacterial activity (*A*)	% growth inhibition (CFU)
1	3.50 ± 0.57 a	99.81 ± 0.12	3.42 ± 0.19 a	99.97 ± 0.02
3	2.97 ± 0.24 a	99.91 ± 0.03	2.44 ± 0.18 a	98.98 ± 0.18
5	2.34 ± 0.33 a	99.43 ± 0.27	2.61 ± 0.54 a	99.26 ± 0.64

a Data are mean values ± SE of three independent experiments. Different letters in antibacterial activity indicate significant differences within each bacterial species. One-way ANOVA, *p* ≤ 0.05, followed by Tukey’s multiple comparisons test.

### 3.5 Scanning Electron Microscopy Micrographs of t-Control and t-MPIA-Van-100 before and after Bacterial Infection

**Figure 3:**
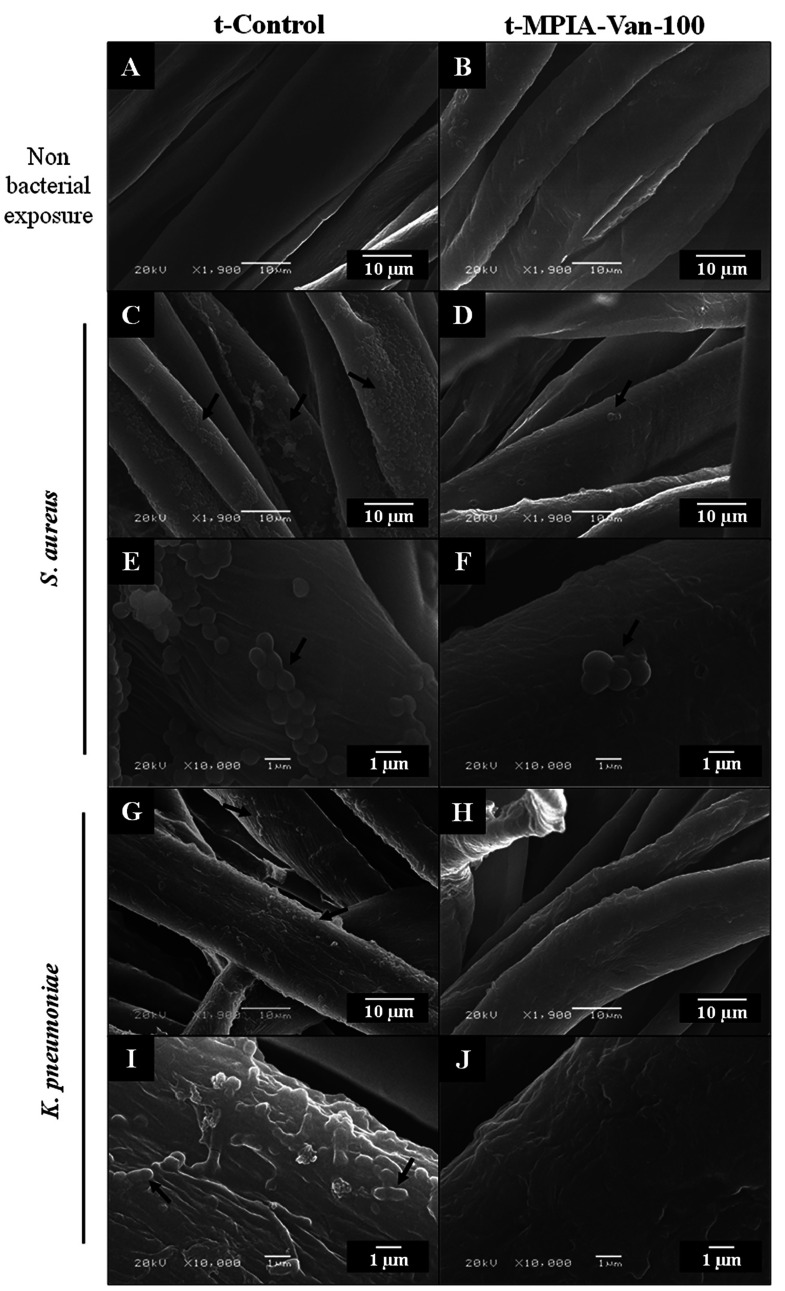
Scanning electron microscopy micrographs of t-Control and t-MPIA-Van-100. Micrographs of t-Control and t-MPIA-Van-100 not exposed to bacteria (A, B) and after exposure to *S. aureus* (C–F) and *K. pneumoniae* (G–J). Black arrows indicate bacteria presence.

To evaluate and confirm the antibacterial capacity of the t-MPIA-Van-100 fabric, scanning electron microscopy (SEM) micrographs were obtained both before and after incubating the samples with *S. aureus* and *K. pneumoniae* for 24 h. As depicted in Figure 3, *S. aureus* and *K. pneumoniae* microorganisms inoculated into t-Control textile are capable of attaching, growing and developing biofilms (as indicated by the black arrows) along the cotton fibers compared to the fabric non exposed to bacteria (Figure 3A,C,E,G,I). In addition, *S. aureus* exhibits a rounded and small morphology (cocci), with an approximate diameter of 1 μm. These entities are observed to configure themselves in clusters or small chains (Figure 3C–F). On the other hand, *K. pneumoniae* displays a rod-shaped morphology ranging between 0.5 and 2 μm and is observed to form aggregates and biofilm structures (Figure 3G,I,J). Furthermore, in the t-MPIA-Van-100, it is challenging to locate microorganisms following bacterial exposure, with only isolated cells present in the case of *S. aureus* and none in the case of *K. pneumoniae* (Figure 3B,D,F,H,J). These results indicate that this fabric effectively inhibited bacterial growth and biofilm formation, consistent with observations from previous experiments. Additional images can be observed in Supporting Information, section S4, Figure S5.

### 3.6 Skin Irritation Assays

Skin irritation was assessed on the Reconstructed Human Epidermis (RhE) by EpiDerm Skin Irritation Test (EPI-200-SIT) (MatTek), a test compliant with the OECD Test Guideline (TG) No. 439 for testing of chemicals. Following the guidelines, RhE tissues were exposed to t-Control and t-MPIA-Van-100 disks in triplicate for 1 h and continued to 24 h postincubation without the materials (see materials and methods).

**Figure 4:**
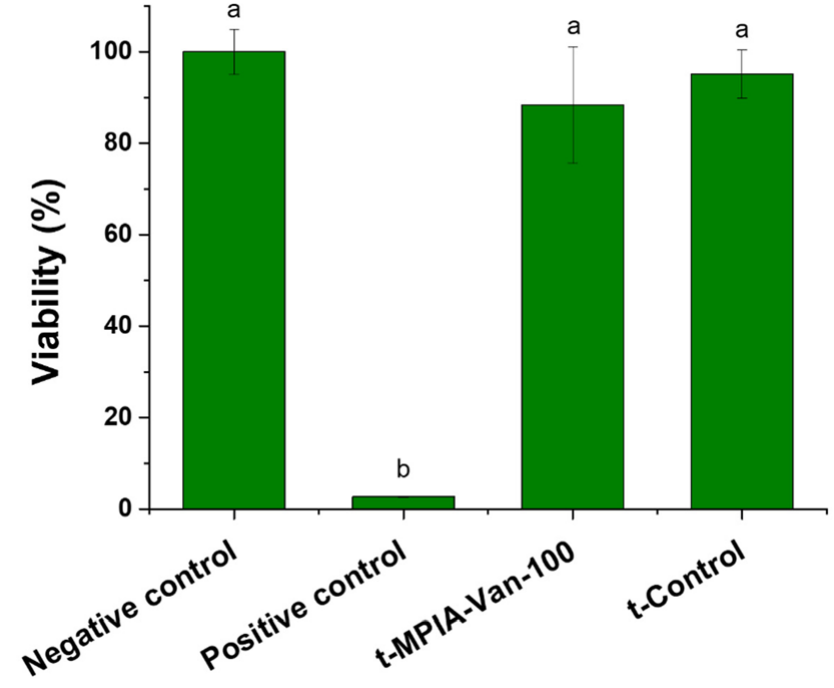
Skin irritation test of the t-Control and t-MPIA-Van-100 textiles. EpiDerm tissues were exposed to t-Control and t-MPIA-Van-100 textiles for 1 h. The tissue viability was evaluated by MTT assay, and it is expressed as a percentage of negative control. Data represented the mean ± SE of 3 independent replicates. Differences were established using a one-way ANOVA followed by Tukey’s multiple comparisons test (*p* ≤ 0.05). The same letter indicates no significant differences between treatments.

According to the OECD guidelines, irritancy was determined by an MTT assay. As defined in EU and Globally Harmonized System of Classification and Labeling Chemicals, GHS (R38/Category 2 or no label), an irritant is predicted if the mean relative tissue viability of three individual tissues exposed to the study substance is reduced below 50% of the mean viability of the negative controls (tissues treated with DPBS). As shown in Figure 4, cotton textiles did not reduce RhE viability at levels lower 50%. Therefore, they could be considered as nonirritant materials. EpiDerm Skin Irritation Test employs reconstructed human epidermis, constituting a three-dimensional evaluation where the test materials are applied directly to the tissue without dilution or modification. As a result, the obtained results maintain a high degree of comparability with *in vivo* conditions, a characteristic not shared by other cytotoxicity assays conducted in 2D cell cultures.

## 4 Conclusions

This study successfully showcased the scalable, practical, metal-free, and cost-efficient feasibility of producing bactericidal aramids derived from parent aramids containing amino groups. The incorporation of vanillin moieties onto aramid coatings in textiles, rich in amino groups, resulted in materials endowed with potent bactericidal properties. Specifically, when the initial aramid possessed 100% amino groups, the vanillin functionalization not only imparted a robust antibacterial effect for *K. pneumoniae* and *S. aureus* as per standard evaluations but also exhibited no skin irritation and reusability. The characterization of the bactericide films as high-performance materials in terms of thermal and mechanical performance revealed that the newly developed bactericidal aramids exhibit thermal performance comparable to that of commercial MPIA and improved Young’s modulus. These findings suggest that the developed materials are well-suited for applications requiring both high performance and bacterial protection. Particularly, these advanced materials could find utility in scenarios where regular washing is impractical or in environments with elevated moisture levels where bacteria tend to proliferate. The unique combination of high performance and bactericidal properties positions these materials as promising candidates for diverse applications, including textiles for first responders’ garments and industrial filters.

## Data Availability

The raw/processed data required to reproduce these findings can be found at http://hdl.handle.net/10259/8385.

## Supporting Information Available

Additional analysis regarding the FTIR and Raman spectra, TGA and DSC thermograms, studies of antibacterial activity, and additional SEM images (PDF)
