# Supplementary material for: High-Performance Aramids with Intrinsic Bactericide Activity
Source: ACS Appl Mater Interfaces. 2024 Feb 7;16(7):9293–302. doi: 10.1021/acsami.3c17919 (PMC10895584; doi:10.1021/acsami.3c17919)
Supplement: Supplementary file 1 — am3c17919_si_001.pdf [file am3c17919_si_001.pdf]

# Supporting Information

# HIGH-PERFORMANCE ARAMIDS WITH INTRINSIC BACTERICIDE ACTIVITY

Sandra de la Parra[2†], Álvaro Miguel[1,4†], Natalia Fernández-Pampín[2], Carlos Rumbo[2], José M. García[1], Ana Arnaiz[1,3]*, Miriam Trigo-López[1]*.

[1] Departamento de Química, Facultad de Ciencias, Universidad de Burgos, Plaza de Misael Bañuelos s/n, 09001 Burgos, Spain.

[2] International Research Center in Critical Raw Materials for Advanced Industrial Technologies (ICCRAM), R&D Center, Universidad de Burgos, Plaza de Misael Bañuelos s/n, 09001 Burgos, Spain

[3] Universidad Politécnica de Madrid, Calle Ramiro de Maeztu, 7, 28040 Madrid, Spain

[4] Facultad de Ciencias, Universidad Autónoma de Madrid, Calle Francisco Tomás y Valiente 7, Fuencarral-El Pardo, 28049 Madrid, Spain

[†] Authors contributed equally to this work

*Corresponding author E-mail: mtrigo@ubu.es, anaaa@ubu.es

**Table of contents**

*S1. Structural characterization of the films*……………………………………………… 3

*S2. Thermal performance of the films*……………… ………………………………… 4

*S3. Antibacterial activity of the textiles*………………………………………………… 5

*S4. Scanning electron microscopy micrographs*…………………………………..….8

## S1. Structural characterization of the films

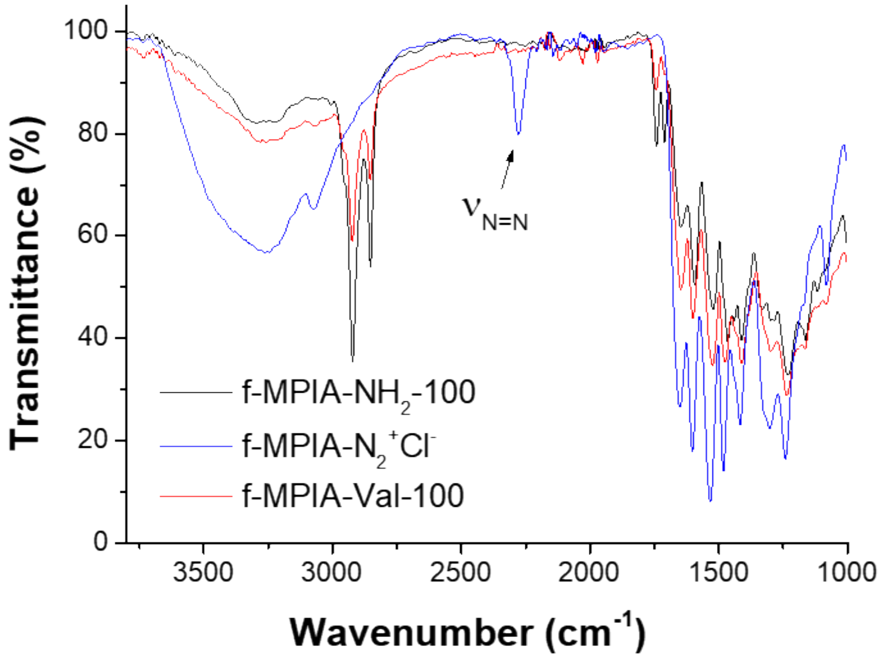


**Figure S1.** FTIR spectra of the films f-MPIA-NH$_2$-100 and f-MPIA-Val-100 and the intermediate upon reaction with sodium nitrite (f-MPIA-N$_2^+$Cl$^-$).

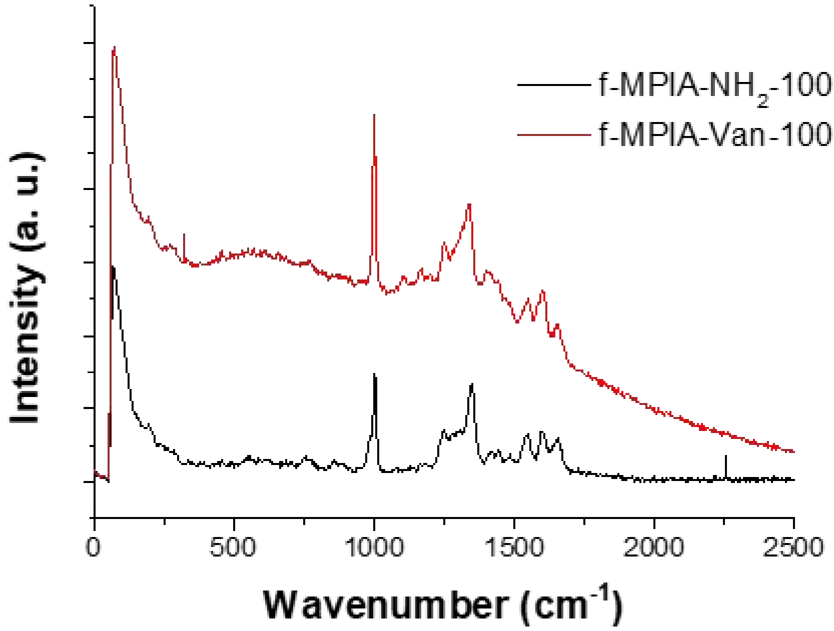


**Figure S2.** Raman spectra of f-MPIA-NH$_2$-100 and f-MPIA-Van-100.

## S2. Thermal performance of the films

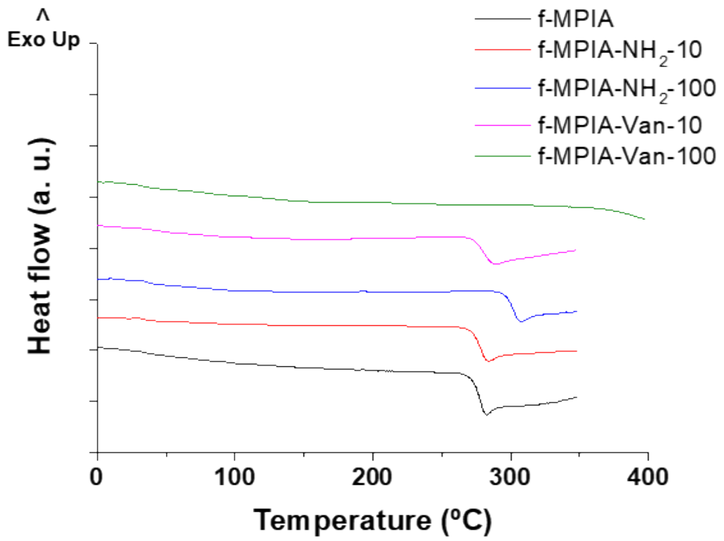


**Figure S3.** Differential scanning calorimetry (DSC) of the films

**a)**

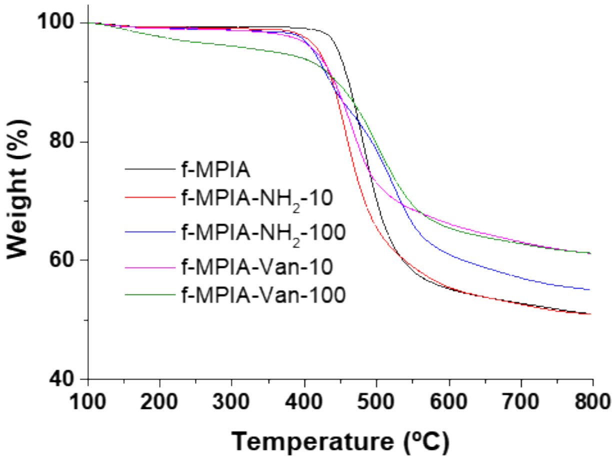


**b)**

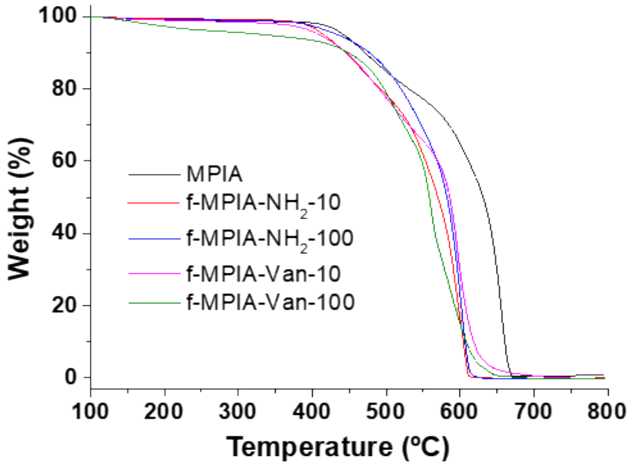


**Figure S4.** Thermogravimetric analysis of the films under a) nitrogen atmosphere and b) synthetic air.

## S3. Antibacterial activity of textiles

**Table S1.** Analyses of the antibacterial capacity and efficacy of t-MPIA (UNE-EN ISO 20743:2022).

| **Strain** | ***S. aureus* WDCM 00193** | | ***K. pneumoniae* WDCM 00192** | |
|---|---|---|---|---|
| **Initial inoculum** | $4.4 \times 10^5$ | | $5.6 \times 10^5$ | |
| **Incubation time** | 20 h | | 20 h | |
| ***Log* differences among the extreme values observed in three samples of the control textile (condition: < 1)** | **0 h** | **20 h** | **0 h** | **20 h** |
| | 0.3 | 0.7 | 0.1 | 0.3 |
| ***Log* Differences among the extreme values observed in three samples of the treated textile (condition: < 2)** | **0 h** | **20 h** | **0 h** | **20 h** |
| | 0.4 | 0.1 | 0.1 | 0.3 |
| **Growth value F (F= lg $C_t$ – lg $C_0$)** | 3.95 (lg $C_t$: 8.94, lg $C_0$: 4.99) | | 3.57 (lg $C_t$: 8.85, lg $C_0$: 5.28) | |
| **Growth value G (G= lg $T_t$ – lg $T_0$)** | 3.99 (lg $T_t$: 9.02, lg $T_0$: 5.03) | | 3.34 (lg $T_t$: 8.81, lg $T_0$: 5.47) | |
| **Antibacterial activity value (A = F – G)** | -0.04 | | 0.23 | |

**Table S2.** Analyses of the antibacterial capacity and efficacy of t-MPIA-$NH_2$-10 (UNE-EN ISO 20743:2022).

| **Strain** | ***S. aureus* WDCM 00193** | | ***K. pneumoniae* WDCM 00192** | |
|---|---|---|---|---|
| **Initial inoculum** | $4.4 \times 10^5$ | | $5.6 \times 10^5$ | |
| **Incubation time** | 20 h | | 20 h | |
| ***Log* differences among the extreme values observed in three samples of the control textile (condition: < 1)** | **0 h** | **20 h** | **0 h** | **20 h** |
| | 0.3 | 0.7 | 0.1 | 0.3 |
| ***Log* Differences among the extreme values observed in three samples of the treated textile (condition: < 2)** | **0 h** | **20 h** | **0 h** | **20 h** |
| | 0.3 | 0.2 | 0.2 | 0.4 |
| **Growth value F (F= lg $C_t$ – lg $C_0$)** | 3.95 (lg $C_t$: 8.94, lg $C_0$: 4.99) | | 3.57 (lg $C_t$: 8.85, lg $C_0$: 5.28) | |
| **Growth value G (G= lg $T_t$ – lg $T_0$)** | 3.99 (lg $T_t$: 9.03, lg $T_0$: 5.04) | | 3.34 (lg $T_t$: 8.77, lg $T_0$: 5.40) | |
| **Antibacterial activity value (A = F – G)** | -0.04 | | 0.19 | |

**Table S3.** Analyses of the antibacterial capacity and efficacy of t-MPIA-$NH_2$-100 (UNE-EN ISO 20743:2022).

| Strain | *S. aureus* **WDCM 00193** | | *K. pneumoniae* **WDCM 00192** | |
|---|---|---|---|---|
| **Initial inoculum** | $4.4 \times 10^5$ | | $5.6 \times 10^5$ | |
| **Incubation time** | 20 h | | 20 h | |
| ***Log* differences among the extreme values observed in three samples of the control textile (condition: < 1)** | **0 h** | **20 h** | **0 h** | **20 h** |
| | 0.3 | 0.7 | 0.1 | 0.3 |
| ***Log* Differences among the extreme values observed in three samples of the treated textile (condition: < 2)** | **0 h** | **20 h** | **0 h** | **20 h** |
| | 0.1 | 0.3 | 0.1 | 0.2 |
| **Growth value F (F= lg $C_t$ – lg $C_0$)** | 3.95 (lg $C_t$: 8.94, lg $C_0$: 4.99) | | 3.57 (lg $C_t$: 8.85, lg $C_0$: 5.28) | |
| **Growth value G (G= lg $T_t$ – lg $T_0$)** | 3.80 (lg $T_t$: 9.02, lg $T_0$: 5.22) | | 3.22 (lg $T_t$: 8.73, lg $T_0$: 5.51) | |
| **Antibacterial activity value (A = F – G)** | 0.15 | | 0.35 | |

**Table S4.** Analyses of the antibacterial capacity and efficacy of t-MPIA-Van-10 (UNE-EN ISO 20743:2022).

| Strain | *S. aureus* **WDCM 00193** | | *K. pneumoniae* **WDCM 00192** | |
|---|---|---|---|---|
| **Initial inoculum** | $4.4 \times 10^5$ | | $5.6 \times 10^5$ | |
| **Incubation time** | 20 h | | 20 h | |
| ***Log* differences among the extreme values observed in three samples of the control textile (condition: < 1)** | **0 h** | **20 h** | **0 h** | **20 h** |
| | 0.3 | 0.7 | 0.1 | 0.3 |
| ***Log* Differences among the extreme values observed in three samples of the treated textile (condition: < 2)** | **0 h** | **20 h** | **0 h** | **20 h** |
| | 0.3 | 0.7 | 0.1 | 0.4 |
| **Growth value F (F= lg $C_t$ – lg $C_0$)** | 3.95 (lg $C_t$: 8.94, lg $C_0$: 4.99) | | 3.57 (lg $C_t$: 8.85, lg $C_0$: 5.28) | |
| **Growth value G (G= lg $T_t$ – lg $T_0$)** | 2.65 (lg $T_t$: 7.93, lg $T_0$: 5.28) | | 2.70 (lg $T_t$: 8.56, lg $T_0$: 5.86) | |
| **Antibacterial activity value (A = F – G)** | 1.30 | | 0.87 | |

**Table S5.** Analyses of the antibacterial capacity and efficacy of t-MPIA-Van-100 (UNE-EN ISO 20743:2022).

| **Strain** | ***S. aureus*** **WDCM 00193** | | ***K. pneumoniae*** **WDCM 00192** | |
|---|---|---|---|---|
| **Initial inoculum** | $4.4 \times 10^5$ | | $5.6 \times 10^5$ | |
| **Incubation time** | 20 h | | 20 h | |
| ***Log*** **differences among the extreme values observed in three samples of the control textile (condition: < 1)** | **0 h** | **20 h** | **0 h** | **20 h** |
| | 0.3 | 0.7 | 0.1 | 0.3 |
| ***Log*** **Differences among the extreme values observed in three samples of the treated textile (condition: < 2)** | **0 h** | **20 h** | **0 h** | **20 h** |
| | 0.2 | 1.0 | 0.1 | 0.8 |
| **Growth value F (F= lg $C_t$ – lg $C_0$)** | 3.95 (lg $C_t$: 8.94, lg $C_0$: 4.99) | | 3.57 (lg $C_t$: 8.85, lg $C_0$: 5.28) | |
| **Growth value G (G= lg $T_t$ – lg $T_0$)** | -0.36 (lg $T_t$: 4.80, lg $T_0$: 5.16) | | 0.15 (lg $T_t$: 5.41, lg $T_0$: 5.26) | |
| **Antibacterial activity value (A = F – G)** | 4.31 | | 3.42 | |

## S4. Scanning electron microscopy micrographs

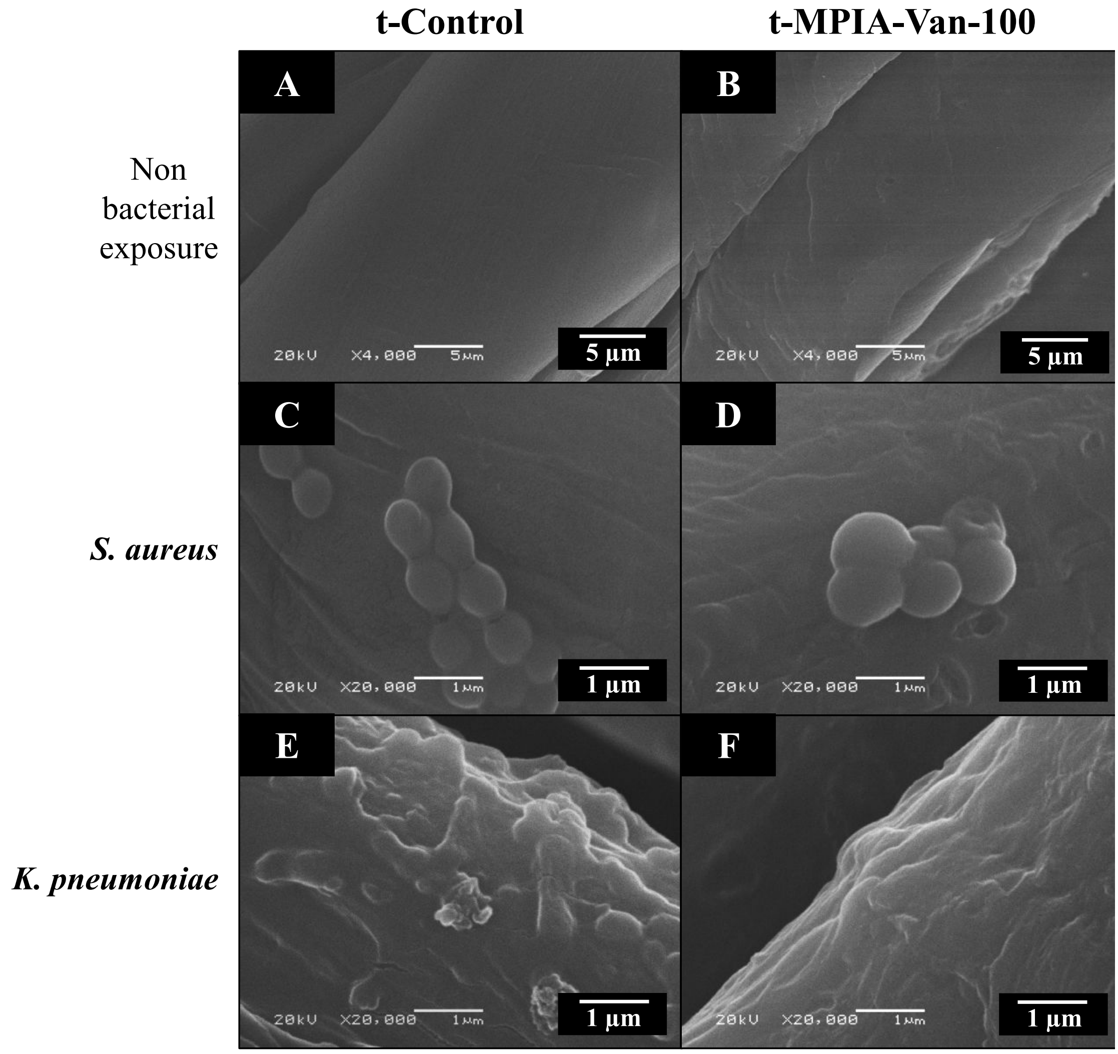


**Figure S5**. Scanning electron microscopy micrographs of t-Control and t-MPIA-Van-100. Micrographs of t-Control and t-MPIA-Van-100 non exposed to bacteria (A, B) and after exposed to *S. aureus* (C, D) and *K. pneumoniae* (E, F).
